# Sunstroke

**Published:** 1887-01

**Authors:** 


Sunstroke.—Dr. C. H. Hughes, of St. Louis, Mo., directs special attention to the great value of bromide of potassium by mouth or by rectum in the treatment of sunstroke. He gives from sixty to one hundred and twenty grains during the first hour, and sixty grains every hour or thirty grains every half hour, largely diluted in peppermint water; sulphuric ether freely to head and spine, and fanned away till six ounces are used; ice at the same time to arms, wrists, abdomen, over the heart, legs, etc., and, in extreme cases of comatose collapse, ice-cold water into the bowels with ginger and capsicum, but ordinarily moderately cold water with two hundred grains of bromide of potassium.—*Weekly Med. Review.*

